# A rapid and inexpensive labeling method for microarray gene expression analysis

**DOI:** 10.1186/1472-6750-9-97

**Published:** 2009-11-25

**Authors:** Mario Ouellet, Paul D Adams, Jay D Keasling, Aindrila Mukhopadhyay

**Affiliations:** 1 The Joint Bioenergy Institute, Lawrence Berkeley National Laboratory, Emeryville, USA; 2 Physical Biosciences Division, Lawrence Berkeley National Laboratory, Berkeley, USA; 3 Department of Chemical Engineering, University of California, Berkeley, USA; 4 Department of Bioengineering, University of California, Berkeley, USA

## Abstract

**Background:**

Global gene expression profiling by DNA microarrays is an invaluable tool in biological research. However, existing labeling methods are time consuming and costly and therefore often limit the scale of microarray experiments and sample throughput. Here we introduce a new, fast, inexpensive method for direct random-primed fluorescent labeling of eukaryotic cDNA for gene expression analysis and compare the results obtained on the NimbleGen microarray platform with two other widely-used labeling methods, namely the NimbleGen-recommended double-stranded cDNA protocol and the indirect (aminoallyl) method.

**Results:**

Two total RNA samples were labeled with each method and hybridized to NimbleGen expression arrays. Although all methods tested here provided similar global results and biological conclusions, the new direct random-primed cDNA labeling method provided slightly better correlation between replicates compared to the other methods and thus increased ability to find statistically significant differentially expressed genes.

**Conclusion:**

The new direct random-primed cDNA labeling method introduced here is suitable for gene expression microarrays and provides a rapid, inexpensive alternative to existing methods. Using NimbleGen microarrays, the method produced excellent results comparable to those obtained with other methods. However, the simplicity and cost-effectiveness of the new method allows for increased sample throughput in microarray experiments and makes the process amenable to automation with a relatively simple liquid handling system.

## Background

DNA microarrays allow global profiling of nucleic acid sequences and have become an important and ubiquitous tool in biological and biomedical research. Although many applications of DNA microarrays have been developed in the past decade [1,2], differential gene expression profiling remains the most widely used application of this technology. Improvements in microarray design now allow rapid fabrication of custom microarrays, representation of an increasingly large number of features on a single glass slide and hybridization of multiple samples on physically separated arrays on the same slide. Robots designed specifically for DNA and RNA extraction are also commercially available now and can considerably reduce the hands-on time required for RNA preparation for microarray studies. Although identification of the most biologically relevant information from a microarray experiment and interpretation of this information in a biological context can be challenging, methods and tools for microarray data analysis have become more widely available and easy to use, and are now streamlining the first step of data analysis. However, the sample labeling procedure remains a rate-limiting step in high throughput microarray workflows.

Several methods to fluorescently label cDNA for gene expression have been developed over the years (reviewed in [3] and [4]). The first method introduced was the direct incorporation of fluorophore-conjugated nucleotides during reverse-transcription (RT). However, this method suffered from lower cDNA yields and significant dye bias (in two-color experiments) due to steric hindrance of the large fluorescent moieties attached to the labelled nucleotides. An indirect method of cDNA labeling, where modified (i.e. aminoallyl) nucleotides are incorporated into the cDNA and chemically coupled with the fluorescent dye post RT, was developed to overcome these shortcomings. This indirect method provided increased dye incorporation and mitigated dye bias, and has become a benchmark for microarray sample labeling, especially in dual labeling experiments. However, this method increased the sample preparation time and cost significantly. Other "indirect" labeling methods were also developed, mainly aimed at increasing specific fluorescence of the labelled product (and conversely, permitting the use of lower amounts of starting material) (e.g. DNA dendrimers), but these methods are still not widely used. Instead, template (RNA) amplification methods, mostly based on an early *in vitro *transcription method [5], coupled with traditional downstream labeling methods, are more broadly adopted when the amount of available RNA is limited, notably because of the efficiency and robustness of the process, as well as the great flexibility it provides regarding the amount of input RNA needed. More recently, NimbleGen introduced a new labeling method based on double-stranded cDNA synthesis followed by labeling with a DNA polymerase by extension of 5'-labeled random primers [6]. This method is very robust in that the yield of each step is excellent and it produces an abundance of labeled material. However it is costly and requires the most time to perform.

We sought a method for fluorescently labeling cDNA for microarray analysis that would be rapid to perform, limit the need for manual handling and reduce the cost significantly when the RNA input is not limiting. In this study we demonstrate a new one-step labeling method--the direct, random-primed cDNA labeling method (hereafter referred to as the direct random method), based on the elongation of 5'-labeled random DNA nonamers during reverse transcription of eukaryotic total RNA. We demonstrate the suitability of our method for gene expression analysis by comparing results with those obtained using the indirect and the NimbleGen-recommended ds-cDNA protocols.

## Results and discussion

### Overview of labeling methods, cDNA yield and dye incorporation

**Figure 1:**
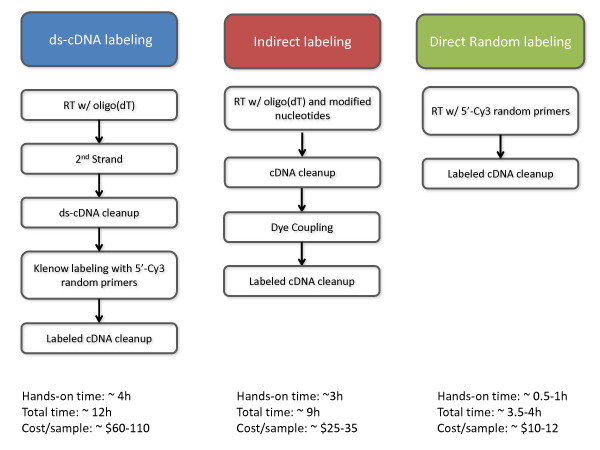
**Overview of the sample labeling methods used in this study**.

Our new direct random-primed labeling method consists essentially of a RT reaction with 5'-labeled random nonamers followed by chemical hydrolysis of the RNA template and silica-based cDNA clean up (in order to remove non-elongated primers and other RT reaction components). This method provides a rapid and inexpensive protocol for sample labeling (Figure 1). In order to evaluate the microarray results obtained with this method, we labelled two *S. cerevisiae *total RNA samples with the NimbleGen-recommended double-stranded cDNA method, the indirect (aminoallyl) method and our direct random method, each in triplicate. The three methods, being intrinsically different, produced different cDNA labeling in terms of representation, dye incorporation, cDNA yield (Table 1) and size distribution (not shown). It is important to note that, because of these differences, it is hard to compare these values directly and predict behaviour in microarray hybridization. For example, our direct random method also reverse-transcribes the ribosomal and other non-coding RNA species, which constitute the vast majority of the total RNA but are not of much interest in a differential expression experiment, and produced cDNA with the lowest dye incorporation. In contrast, the indirect method produced cDNA of the largest median size and spread and exhibiting the highest dye incorporation, but the cDNA yield was the lowest. Finally, the ds-cDNA procedure produced the most labeled cDNA, roughly six times the recommended amount for a microarray hybridization, with a median cDNA size consistent with random priming. Of note, the random-primed Klenow labeling reaction uses ds-cDNA generated with an oligo(dT)-primed RT reaction as template and contains the sense strand, which does not hybridize to the microarray. The higher specific dye incorporation in the ds-cDNA method than in our direct random protocol is likely due to the amount of 5'Cy3 random nonamers used in each reaction, with a mass ratio of primer to template of about 33 and 0.7, respectively.

**Table 1:** cDNA yield, dye incorporation and amount of material used for hybridization (*n *= 6 for each method).

	**Labeling Yield (avg. ± S.D.)**			**Quantity hybridized**
		
	**cDNA** **(μg)**	**Dye** **(pmol)**	**Ratio** **(pmol dye/μg cDNA)**	**cDNA (μg)**
	
**ds-cDNA**	25.8 ± 0.8	463 ± 66	18 ± 3	4.00
**Indirect**	0.33 ± 0.06	33 ± 5	105 ± 26	0.30
**Direct Random**	7.7 ± 0.6	85 ± 6	11.1 ± 0.5	5.00

S.D., standard deviation.

### Concordance of microarray results

Despite the lower dye incorporation in the cDNA using our direct random method, we decided to hybridize the samples to NimbleGen 4-plex expression microarrays. For each labeling method, different amounts of labeled cDNA were hybridized to the arrays (Table 1). Since NimbleGen recommends hybridization of 4 μg of labeled ds-cDNA[6], we used this quantity for the ds-cDNA method. In the absence of guidelines for the other two labeling methods, we hybridized an amount of cDNA consistent with the respective yield of each method (Table 1). Visual inspection of the resulting slide images revealed differences in the global fluorescence intensity of individual arrays (not shown), the brightest arrays being achieved with the ds-cDNA method whereas the other two methods produced a similar but slightly lower global fluorescence. To compensate for these differences, the arrays were scanned independently in order to adjust the photomultiplier tube (PMT) gain for each array as recommended [6].

Pair-wise correlation of the intensities obtained with each labeling method after summarization and normalization were examined for each set of replicates for each sample (*n *= 3) and averaged (*n *= 6) (Table 2). All three methods produced very good correlation of internal replicates, but the highest average correlation and the narrowest spread of values was obtained with our direct random method. However, the average correlation coefficient fell dramatically when comparing the different labeling methods (Table 2), the ds-cDNA method being the most different from the other two methods. This result indicated there were differences in the cDNA population produced by the different labeling methods, which caused different hybridization characteristics. Nonetheless, since each method is very reproducible (Table 2), it is expected that the relative changes in expression deduced using any of these methods would be similar.

**Table 2:** Average pair-wise correlation coefficients (± S.D.) of normalized intensities of replicate arrays within a labeling method (*n *= 6) and across methods (*n *= 18).

	Direct Random	Indirect	ds-cDNA
**Direct Random**	0.995 ± 0.001	-	-
**Indirect**	0.934 ± 0.006	0.990 ± 0.004	-
**ds-cDNA**	0.62 ± 0.02	0.55 ± 0.02	0.987 ± 0.002

**Figure 2:**
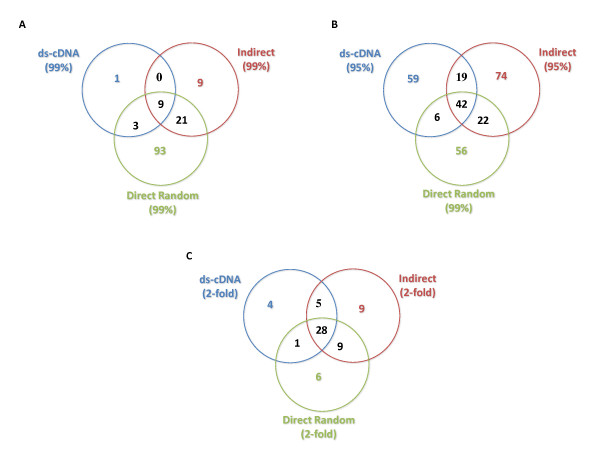
**Overlap of differentially expressed genes found by each labeling method at different confidence intervals**.

**Figure 3:**
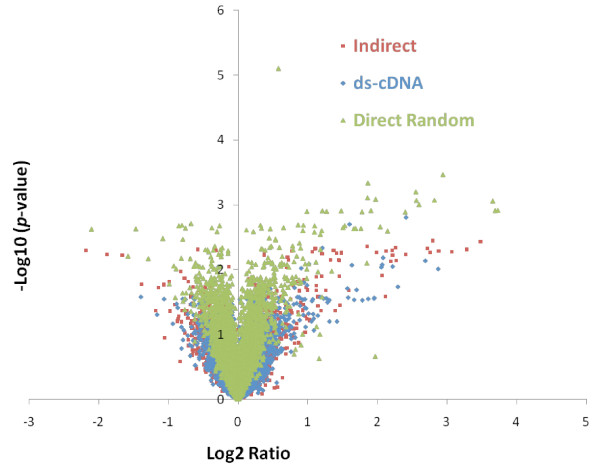
**Distribution of *p*-values**.

Interestingly, the direct random method revealed the most differentially expressed genes at any given significance level (Table 3), and most of the genes with a fold-change of at least 2 (in any given direction) were found at 99% confidence. This was not the case with the other two methods (Table 3). Lowering the significance level to 95% increased the number of genes found by each method considerably regardless of the fold-change. Similar numbers of differentially expressed genes were found when comparing the direct random method at a significance level of 99% with the other two methods at 95% (Table 3). The direct random method found the largest number of significantly differentially expressed genes (126) at 99% confidence (136 different genes total across all three methods) (Figure 2A). Very few differentially expressed genes were found exclusively by either the indirect or the ds-cDNA methods (Figure 2A), whereas many such changers were uniquely found by the direct random method. Most of the differentially expressed genes unique to the direct random method were found to have a modest (< 2) fold-change (Table 3). Overall, the median fold-change (in any direction) of the genes found at 99% confidence was 4.15 for the ds-cDNA method, 2.79 for the indirect method and 1.56 for the direct random method. The smaller median fold-change of genes changing with high confidence suggests that the direct random method generally provided higher statistical confidence in lower fold-change values. These results can be explained by the higher correlation between replicates obtained with this method (Table 2), which translate into better *p*-values on the fold-change determined, especially for genes with smaller fold-change (Figure 3). This could possibly be due to the fewer manipulations in the direct random method compared to the other two methods, reducing experimental variability and providing greater precision. However, it is difficult to determine if these genes are really changing or are the result of a loss of specificity. The large representation of the rRNA species in the direct random product can potentially cause non-specific hybridization. Although the probe design and hybridization conditions used should provide the required specificity, additional controls, such as a large number of array features with random sequences, and samples, e.g. depleted of rRNA or purified mRNA, would have been necessary to assay and compare specificity of the methods and determine more appropriate statistical cut-offs.

**Table 3:** Number of statistically significant differentially expressed genes at different confidence intervals.

	99%	95%	90%
**ds-cDNA**	13 (11)	126 (32)	329 (38)
**Indirect**	39 (30)	157 (48)	385 (50)
**Direct Random**	126 (37)	653 (40)	1242 (42)

In parentheses, number of genes with a fold-change greater than 2 (in any direction) in this category.

In order to analyze comparable numbers of differentially expressed genes for the three methods, we chose a 95% confidence interval for the data from the indirect and ds-cDNA methods and 99% confidence interval for the direct random (Table 3 and Figure 2B). Using these criteria, a similar proportion (53-56%) of differentially expressed genes found by any method was also found by other methods (Figure 2B). Overlap between the methods increases substantially when comparing the genes with the largest fold-change values (> 2), most of which were found by more than one method (Figure 2C).

**Figure 4:**
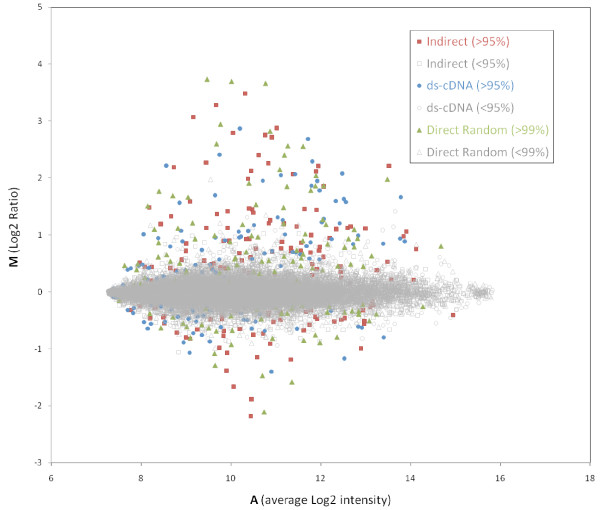
**MA plot**. The statistical cut-offs used for each series are shown in parentheses.

However, it is possible that any given threshold may introduce a bias as to which genes are represented in each group. In order to more accurately compare the global correlation between the fold-change values obtained with each method, we omitted a statistical cut off for the following comparisons. All three methods agree largely as to the direction of change in gene expression (upregulated or downregulated). 61 out of the 62 genes with a fold-change > 2 with any method were found to be changing in the same direction across all methods. The only gene with inconsistent data across the three methods was also associated with a high *p*-value (> 0.2) in all methods. Overall, 3,297 genes (57.4%) changed in the same direction across all three methods; 99.7% of the genes that don't agree may not be significantly changing (the average fold-change for these genes is 1.00 with a standard deviation of 0.09; their mean *p*-value is 0.6). All three methods produced similar distributions of significantly differentially expressed genes across the range of signal intensities observed (Figure 4).

The correlation of fold-change values across methods is arguably the best way to effectively compare methods because it plays a major role in the selection of gene lists and in the interpretation of the results in a biological context. Furthermore, since each method is very reproducible, it would be expected that the differences between the cDNA populations produced by the each type of labeling would be similar in both control and experimental samples and thus produce similar fold-change values.

**Figure 5:**
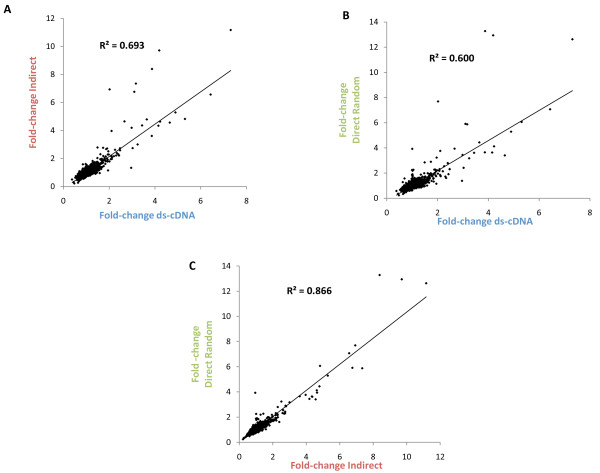
**Correlation of fold-change values obtained with the different labeling methods**. A: indirect labeling *vs*. ds-cDNA methods. B: direct random *vs *ds-cDNA. C: direct random *vs*. indirect.

However, in our dataset, the fold-change values from the indirect method correlated best with those from our direct random method (R^2 ^= 0.87), but the correlations were not as good for the other comparisons (Figure 5). Similar trends were observed even when only genes with fold-change greater than 2 (by any method) were considered (not shown). The modest correlation coefficients of fold-change values obtained when comparing the indirect and the direct random datasets with the ds-cDNA dataset were surprising at first. However, studies comparing microarray results often find relatively low correlations. For example, the MicroArray Quality Control (MAQC) project data shows only 70-85% concordance of qualitative gene calls (presence or absence) across different test sites using the same platform (i.e., a commercial microarray and its recommended labeling method) [7] and correlation coefficients of fold-change values ranging from 0.53 to 0.92 between platforms [8].

**Figure 6:**
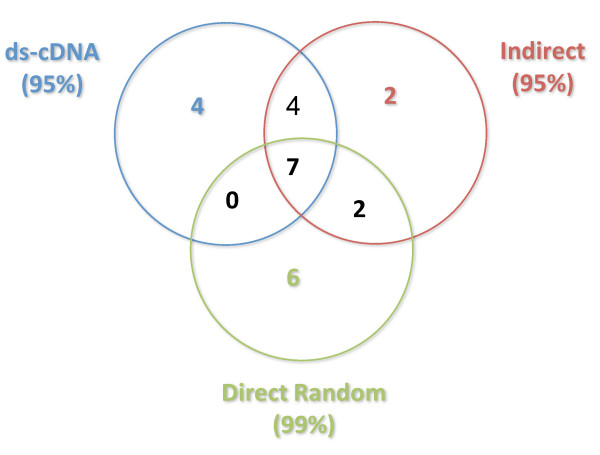
**Overlap of the 15 GO categories (with less than 500 genes) with the most significantly differentially expressed genes for the three labeling methods**. The statistical cut-offs used to generate the lists of differently expressed genes for each method are shown in parentheses.

Despite the specific differences in measured fold-change (and statistical confidence in these measurements) across labeling methods, the results are globally similar and suggest that the biological interpretation and conclusions from these experiments would be similar. To assess this hypothesis, we examined gene ontology categories. First, since we used an experimental system very similar to the one used in an experiment published elsewhere [9], we examined categories expected to have a number of genes differentially expressed based on this study. All three methods were able to detect a similar number of significantly changing genes in three important categories (Table 4), suggesting similar conclusions from all three methods. We also took an unbiased approach where we compared the 15 GO categories with the most genes differentially expressed genes between labeling methods. Here we omitted GO categories with more than 500 genes in an attempt to avoid the broad, less meaningful categories. Most of the categories found to contain the most differentially expressed genes by one given method were also found by other methods, but each method also found unique categories based on these criteria. The direct random method detected the most unique categories (Figure 6). However, several arbitrary cut offs used here can affect the representation of each category and this data should be considered as indicative at best. Furthermore, we cannot conclude that the unique categories found by any method provided additional biological insight supporting the differences observed without extensive individual validation with a variety of techniques, which is beyond the scope of this work. In general, the results from the three methods were predominantly overlapping, supporting the idea that similar global biological conclusions would be extracted from the data produced by each method.

**Table 4:** Number of differentially expressed genes in selected GO categories.

	Direct Random (99% - 126 genes)	Indirect (95% - 157 genes)	ds-cDNA (95% - 126 genes)
**Response to stress**	15	18	11
**Response to chemical stimulus**	23	23	19
**Transport**	36	34	24

### Comparison with other gene expression technologies

Microarray data can be affected by numerous artefacts, resulting in expansion or compression of fold-change values. To determine if one labeling method provides a more accurate estimate of fold-change over the others, we conducted qPCR analysis on a small set of genes. We selected target genes presenting 1) good *p*-values across labeling methods but different fold-change values, 2) genes found as changing but with different statistical significance levels for different methods, or 3) significantly changing in some cases but not in others. The qPCR results and comparison with microarray data are summarized in Table 5. The qPCR data is generally in agreement with the array data, the best correlation coefficient being obtained with the indirect method (R^2 ^= 0.933), whereas the other two labeling methods correlated similarly less well with the qPCR data (0.85 and 0.83, for the direct random and the ds-cDNA methods, respectively). These correlation values between the array and the qPCR data are similar to the correlations obtained by others between microarray data from different platforms and TaqMan [7] or SYBR Green [10] qPCR data, whereas in another study the mean correlation coefficients ranged from 0.69 to 0.89 [8]. However, it should be noted that we used an oligo(dT) priming strategy for the RT reaction which was then used as a template for the qPCR. Further, the primer design was biased towards the 3'end of the genes. These factors may introduce a bias that improves the correlation between qPCR and microarrays that used oligo(dT)-primed cDNA labeling methods, namely the indirect and the ds-cDNA methods.

**Table 5:** Fold-change data comparison between different microarray labeling methods and qPCR for selected genes.

	Fold-change			
	
Gene	Microarrays			
		
	Direct Random	Indirect	ds-cDNA	qPCR
YNL072W (*RNH201*; Ref1)	1.0	1.0	1.0	1.0
YLR185W (*RPL37a*; Ref2)	1.0	1.1	0.9	1.0
YBL005W (*PDR3*)	2.2**	1.9	2.1	1.6**
YDR011W (*SNQ2*)	3.4**	4.2*	3.0*	4.6**
YDL020C (*RPN4*)	1.9**	1.8*	1.7	3.6**
YFR003C (*YPI1*)	1.0	1.1	0.8**	1.0
YGR035C	12.6**	11.7**	7.3**	10.8**
YGR052W (*FMP48*)	3.2**	3.0*	3.2*	2.9**
YGR236C (*SPG1*)	0.4**	0.4*	0.7*	0.5**
YLR346C	7.7**	6.9**	2.0**	6.2**
YOL156W	12.9**	9.7**	4.2**	6.8**

(* *p*-value < 0.05; ** *p*-value < 0.01)

We also had access to data collected using a new gene expression technology, the NanoStrings nCounter [11], to estimate the relative expression of 205 yeast transcription factors in these RNA samples. The advantages of the nCounter system over other technologies reside in the absence of enzymatic bias and in that the absolute abundance of each transcript is established by counting directly the number of RNA molecules for that transcript in the sample [11]. From this dataset, we used 7 transcription factors found to be significantly differentially expressed (*p*-values < 0.05), and compared the fold-change values for these genes with the data obtained by microarrays for each labeling method (Table 6). Both the indirect and the direct random methods present an excellent correlation coefficient with the nCounter data (0.981 and 0.975, respectively), while the ds-cDNA method's correlation was lower (0.842).

**Table 6:** Fold-change data comparison between different microarray labeling methods and the nCounter technology for select genes.

	Fold-change			
	
Gene	Microarrays			
		
	Direct Random	Indirect	ds-cDNA	nCounter
YBL005W (*PDR3*)	2.17**	1.95	2.07	2.17**
YDL020C (*RPN4*)	1.87**	1.82*	1.7	1.69*
YDL048C (*STP4*)	1.80**	2.03	1.80*	2.00**
YER130C	2.79**	2.30*	2.40*	2.22**
YFL052W	0.84	0.81	0.84	0.75*
YGL209W (*MIG2*)	5.91**	6.76**	3.10**	5.20**
YMR016C (*SOK2*)	1.65*	1.91*	1.69	1.80*

(* *p*-value < 0.05; ** *p*-value < 0.01)

It has been shown that the fold-change values obtained in qPCR are highly dependent on the method used [12], and despite our efforts to use methods considered to be the most accurate, specific genes may still have unpredictable biases making comparison across methods difficult. For example, two genes (*RPN4 *and *PDR3*) present in both the qPCR and the nCounter data sets showed relatively different fold-change values with the two methods (Tables 5 and 6). In the absence of known or expected fold-change values in this data set, it is not possible to assign greater accuracy to any of the methods. Furthermore, both our qPCR and nCounter datasets are very small and have been chosen arbitrarily, and no method has shown a clear superiority over the others in estimating fold-change values. However, all three methods broadly produced similar, reproducible results and would be considered suitable sample preparation protocols for microarray workflows.

## Conclusion

The goal of this work was to introduce a labeling method that is comparable to currently used protocols but reduces sample cost and labeling time. Despite lower dye incorporation and global fluorescence of the array, the new direct random method provided excellent reproducibility across replicates, possibly because of the minimal manipulations. In order to present a generally useful protocol, no attempts were made to try to optimize different parameters in the labeling protocol or the amount of labelled cDNA hybridized to the array. We used a typical number of arrays for a given sample comparison in a large scale screening experiment. Slightly different results could be expected with different source materials, microarray platforms or hybridization conditions and optimization of certain parameters or larger number of replicates may be beneficial for particular systems. However, the successful use of the direct random method with eukaryotic RNA samples suggests that this method would be universally applicable independent of the source of RNA. Furthermore, the method could be adapted for samples of limited abundance such as fixed sections, sorted cells or environmental samples, provided that an RNA amplification step is performed before the labeling. The simplicity of our method also makes it amenable to automation using a relatively simple liquid handling robot for very high throughput microarray applications. In this scheme, 96 RNA samples could be reverse-transcribed, labeled and purified at once, and hybridized to 8 NimbleGen, 12-plex, microarray slides in a single day by one person.

## Methods

### Biological material and RNA extraction

The *S. cerevisiae *strains used in this study are engineered strains EPY330 and EPY338 described previously [9]. Briefly, three independent colonies for each strain were pre-cultured in SD-His-Met-Leu medium and used to inoculate 5 ml of YPG medium [9] for induction at 30°C in culture tubes. Cells were harvested after 24 h of induction by quick centrifugation and immediately frozen in liquid nitrogen, disrupted with glass beads in a bead-beater and total RNA was extracted using the RNeasy Mini kit (QIAGEN), including the on-column DNAse treatment. RNA was quantified by spectrophotometry with a Nanodrop ND-1000 (Thermo Scientific) and its integrity verified on a 2100 Bioanalyzer (Agilent). Equal amounts of RNA from each replicate culture were pooled for each strain and used for microarray labeling and hybridization, qPCR and the nCounter analysis. All gene expression ratios in this paper are expressed as EPY330/EPY338.

### Microarrays

For each labeling reaction, 10 μg of total RNA was used as starting material regardless of the labeling method. For the new direct random method, 7 μg of 5'Cy3 random nonamers (TriLink Biotechnologies, San Diego, CA) were added to the total RNA for a volume of 18.5 μl, heat denatured at 70°C for 5 minutes and placed on ice immediately. The remainder of the RT reaction components (6 μl of 5× First-Strand buffer, 1.5 μl of 0.1 M DTT, 1 μl of 10 mM dNTPs, 2 μl of 400 U/μl SuperScriptIII (Invitrogen) and 1 μl of 40 U/μl RNAseOut) were added and the reaction incubated at 25°C for 5 minutes and at 42°C for 3 h. Template RNA was chemically hydrolysed by addition of 1 volume of a 200 mM NaOH, 20 mM EDTA solution and incubation at 65°C for 10 minutes. The hydrolysis reaction was neutralized with 1 volume of 1 M HEPES, pH 7.0 and the labeled cDNA purified on a Qiaquick column (QIAGEN) following the manufacturer's recommendations for PCR purification. For the ds-cDNA protocol, the labeling method was carried out as recommended by NimbleGen [6], except that the components for the ds-cDNA synthesis were purchased separately and SuperScriptIII was used for the first strand cDNA synthesis. For the indirect method, the SuperScript Plus Indirect cDNA Labeling System (Invitrogen) was used with Alexa Fluor 555 reactive dye (Invitrogen) following the manufacturer's recommendations, except for the chemical hydrolysis of RNA and cDNA purifications, which were carried out as described above for the direct random method with the exception that the kit wash buffer in the first cDNA purification was replaced with 80% ethanol.

cDNA yields and dye incorporation were obtained with the Nanodrop ND-1000, using a factor of 37 for ss-cDNA or a factor of 50 for ds-cDNA. Amounts of cDNA to be hybridized to each array (Table 1) were aliquoted and dried in a SpeedVac (Thermo Scientific). NimbleGen *S. cerevisiae *4-plex expression microarrays (cat. # A6186-00-01) were used, and targets labeled with the different methods were randomly distributed on 5 microarray slides. Hybridization on a 12-bay NimbleGen Hybridization System and array washes were performed as recommended by NimbleGen [6]. Individual array images were acquired independently using a GenePix Professional 4200A scanner (Axon Instruments), adjusting the PMT gain for each image as recommended [6]. Image analysis was performed with the NimbleScan software (Nimblegen), and feature intensities were exported as .pair files. ArrayStar 3.0 (DNASTAR, Madison, WI) was used for probe summarization and normalization (RMA algorithm, quantile normalization), statistical analysis of differentially expressed genes (Student's *t*-test with Benjamini-Hochberg false discovery rate correction) and gene ontology analysis. The entire microarray data set is available at the Gene Expression Omnibus (accession GSE15816).

### Other gene expression measurements

For qPCR, three independent RT reactions were performed for each RNA sample. Briefly, for each reaction, 1 μg of RNA was reverse-transcribed with SuperScriptIII (Invitrogen) using an oligo(dT) primer following the manufacturer's recommendations. The cDNA reactions were treated with RNAse H and diluted 10-fold; 3 μl of the diluted template was used in the qPCR reactions. The PerfeCTa SYBR Green SuperMix (Quanta Biosciences, Gaithersburg, MD) was used as recommended on a StepOnePlus instrument (Applied Biosystems). Primers were designed with the VectorNTi 10 software (Invitrogen) (Tm 60-65°C, primer length 20-25 bases, 40-60% GC, amplicon size 115-175 bp) with a preference towards the 3' end and are listed in Table 7. For each gene, two replicates of each cDNA were run (*n *= 6 for each RNA sample) and the coefficient of variation on the C_T _of replicates was < 1.7% for all replicate measurements. Efficiency of individual PCR reactions was determined with the LinRegPCR tool [13], and efficiency of all reactions with the same target amplicon was averaged as in the (PavrgE)^Ct ^model [14] and used in the Pfaffl equation [15] to obtain efficiency-corrected, normalized relative quantification values. Two genes (YNL072W and YLR185W) were selected as reference genes in this experiment based on the average fold-change across the entire microarray data set (closest to 1); their C_T _and PCR efficiency were averaged for ratio calculations of target genes ran on the same qPCR plate. In order to determine a *p*-value on the gene expression ratio that takes into account the efficiency and the reference gene normalization, we computed a value *N *proportional to the initial amount of template for each replicate qPCR reaction:

where is *E*_*target *_the average PCR efficiency for that target amplicon across all reactions, *E*_*reference *_is the average efficiency of the two reference genes across all replicates, *Ct*_*Target *_is the C_T _obtained for that target gene in a particular replicate, and *Ct*_*reference *_is the average C_T _for the reference genes across all the replicates in that sample. The values *N *for each group of sample replicates were submitted to a Student's *t*-test (2-tailed, independent samples with equal variance) to obtain a *p*-value.

For the nCounter [11] analysis, RNA from each pool was processed in triplicate by NanoStrings Technologies (Seattle, WA) with probes corresponding to 205 *S. cerevisiae *transcription factors. Raw counts were normalized to the average counts for all control spikes in each sample [11] and the normalized counts in the replicate samples were averaged. In this data set, 7 transcription factors were found to be significantly differentially expressed (*p *< 0.05; *t*-test with Benjamini-Hochberg false discovery rate correction) and used for comparison with the microarray data.

**Table 7:** qPCR primers used in this study.

ORF ID	Gene Name	Forward	Reverse
YBL005W	*PDR3*	GCTTCTGCCTCAGCAGCAAACTCA	GGCTAGGCGCAGAATGTTGTCTTTT
YDR011W	*SNQ2*	CACAACCTGTCTCATTGATGCCTGG	TGAGCCGTTTGGTGGGTTGAAGT
YDL020C	*RPN4*	CAGTATCAGCATCAAACTGCCAGCC	CTGGAATCACTTGGTGAGGATGGTG
YFR003C	*YPI1*	ATGATGATGGATCCTCTTCTTCCGG	GTTGGATTTCATAAGCATTGGGGCT
YGR035C	-	CACCAGCCAAGACTACAAGAACGGA	TGATGGGAACTTTGTCTGCATGTGA
YGR052W	*FMP48*	ATCTGATGTTCGGCGATTGCCTT	TGCTGCAGGCTAACGTGTAGGTCTT
YGR236C	*SPG1*	ATATTATGTTAGGGCTGGTGGGCGC	GCCTTAGTTGTGTCTACGCCGTTGA
YJL219W	*HXT9*	AAGTTGTGGCCTCAAGGAAGCAGTC	TTGCCATTCCTCTTGATTTGACCCT
YLR346C	-	TTGCAGAGTGGGTAGCATGTCCATG	TCCTGGGCAGCCTTGAGTAAATCAT
YOL156W	*HXT11*	ATGCTGCTTTGCTGTGTTTGCCTC	TAACGTAACAGCCGCCTGCCCA
YNL072W	*RNH201*	GGACCACCAGCGTCCTATCAGAAGA	TCATCGGGATCCCTCTTCAAGGA
YLR185W	*RPL37a*	CCTGTTCCTCCTGTGGTTATCCAGC	AAGCAGAGCCGGTTTGGAAACC

## Authors' contributions

MO conceptualized the method. MO and AM designed the experiments. MO conducted experiments and analyzed the data. MO and AM wrote the paper. All authors discussed the results and commented on the manuscript.
